# Spontaneous Uterine Rupture of an Unscarred Uterus before Labour

**DOI:** 10.1155/2012/598356

**Published:** 2012-12-03

**Authors:** Mamour Guèye, Magatte Mbaye, Mame Diarra Ndiaye-Guèye, Serigne Modou Kane-Guèye, Abdoul Aziz Diouf, Mouhamadou Mansour Niang, Hannegret Diaw, Jean Charles Moreau

**Affiliations:** ^1^Clinique Gynécologique et Obstétricale, Centre Hospitalier Universitaire Aristide Le Dantec, Avenue Pasteur, BP 3001, Dakar, Senegal; ^2^Centre Hospitalier Régional Heinrich Lübke, BP 278, Diourbel, Senegal

## Abstract

Uterine rupture is a public health problem in developing countries. When it is spontaneous, it occurs most often during labor in a context of scarred uterus. Uterine rupture during pregnancy is a rare situation. The diagnosis is not always obvious and morbidity and maternal and fetal mortality is still high. We report a case of spontaneous uterine rupture during pregnancy at 35 weeks of an unscarred uterus before labour. This is an exceptional case that we observe for the first time in our unit.

## 1. Introduction

Rupture of a pregnant uterus is one of the life-threatening complications encountered in obstetric practice. It is a rare complication in developed countries, but is one of the causes of maternal and perinatal morbidity and mortality in Africa. There are several risk factors associated with rupture of uterus, but the most common is a previous Cesarean section. Rupture of an unscarred uterus is a rare event. We report a case of a complete rupture of the uterus before labor, in a gravid woman who had an unscarred uterus.

## 2. Case

A 37-year-old patient, gravida 5 para 4, at 35 weeks of gestation was admitted to the hospital because of an abdominal pain since 18 hours, and vaginal bleeding. Her general medical history revealed no diseases or allergy. Her obstetrical history obtained by anamnesis and her documents revealed a multipara patient with a history of four pregnancies that ended spontaneously by vaginal delivery. Cesarean section has never been performed. Current pregnancy included 2 prenatal visits without sonographic examination.

The patient was hemodynamically stable without abdominal tenderness or peritoneal signs.

**Figure 1:**
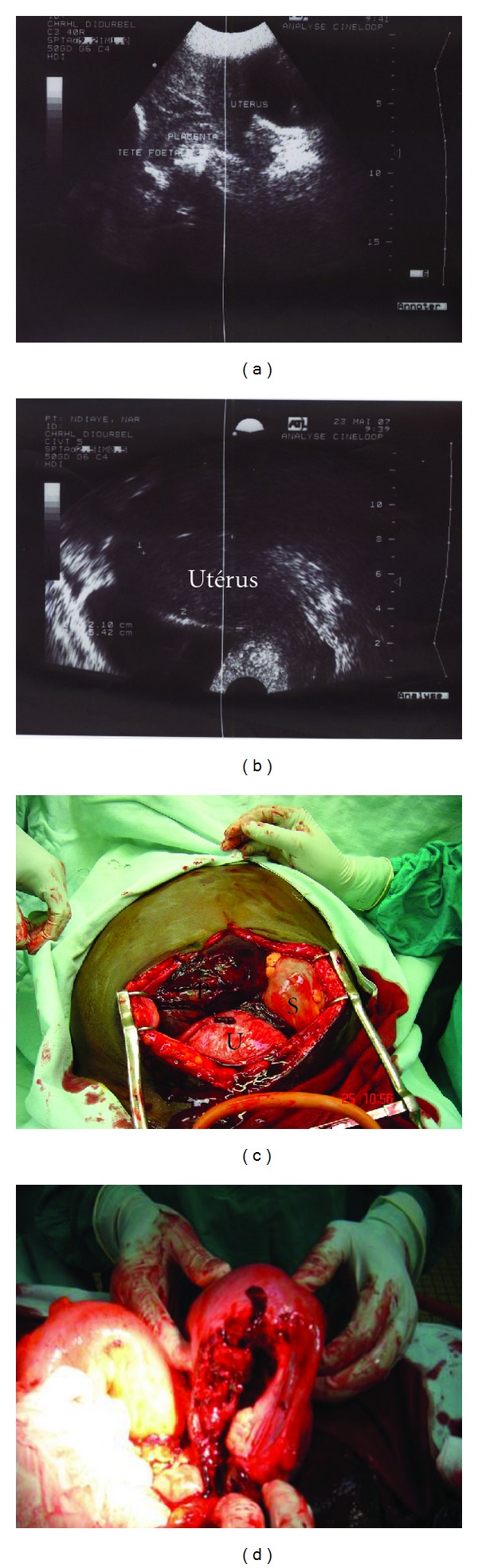
(a) Ultrasound image showing empty uterus, placenta, and fetal head; (b) empty uterus (extension); (c) visualization of the uterus (U), the gestational sac (S), and placenta (P) after opening the abdomen; (d) image showing uterine rupture.

No fetal heart rate was detected. Vaginal examination revealed a closed cervix and no effacement or dilatation. Sonographic examination found an enlarged empty uterus, a fetus in the abdominal cavity, corresponding to 35 weeks of pregnancy (Figures 1(a) and 1(b)). The patient was rushed to the operating room for emergent laparotomy. At the opening of the abdominal wall, the whole intact amniotic sac with fetus inside was protruded into the abdomen (Figure 1(c)). After amniorexis, a male fetus of 2950 g was delivered. Further inspection showed posterolateral uterine rupture interesting the body and extending to the lower transverse segment (Figure 1(d)); the ipsilateral uterine pedicle was intact. Repair of the laceration was not possible. A hysterectomy was performed. No other complications were noticed during the operation and estimation of blood loss was about 1150 mL. The patient received blood transfusion and was discharged after 9 days of postoperative hospitalization without any complications.

## 3. Discussion

Uterine rupture is a common complication of pregnancy in developing countries. However, it is very rare in developed countries. In the USA, the incidence varies between 1 : 8,000–15,000 births. The majority of uterine rupture during pregnancy involves scarred uterus. Rupture of an unscarred uterus is a rare event involving 1 : 17,000–20,000 deliveries [1]. In such cases, rupture may be either traumatic or spontaneous. This frequency is often higher in developing countries, where it can reach 75% of cases in some areas [2]. Clinical signs of uterine rupture during pregnancy are nonspecific and can be confusing. Indeed, it is not always easy to distinguish it with other abdominal emergencies (appendicitis, gallstones, pancreatitis, etc.) [3]. Importance should be given to abdominal pain and digestive disorders. In all cases of abdominal pain in pregnancy, the fetal status must be systematically checked.

The high parity is recognized as major risk factor of spontaneous uterine rupture in unscarred uterus [3]. Other etiological factors classically recognized as contributing to a rupture of unscarred uterus are: obstetric maneuvers, malpresentations especially transverse fetal position, cephalopelvic disproportion, excessive uterine expressions, abnormal placentation (placenta percreta mainly), trauma due to uterine curettage, and uterine abnormalities [2, 4]. In some cases the rupture of gravid uterus has no obvious cause. In his series of 40 uterine ruptures, Schrinsky and Benson [5] found ten spontaneous ruptures without any predisposing factors. The case presented here emphasizes the possibility of uterine rupture, even in women with unscarred uterus and before labour. Besides multiparity, no apparent cause was found.

Early surgical intervention is usually the key to successful treatment of uterine rupture [3]. The therapeutic management is a total or subtotal hysterectomy. The suture can be performed [3] and helps to preserve the reproductive function of patients who have never given birth with a recurrence risk of uterine rupture assessed between 4 and 19% at a subsequent pregnancy [2]. For this reason, it has been recommended that women with a previous uterine rupture undergo an elective Caesarean delivery as soon as fetal lung maturity can be demonstrated [6].

Uterine rupture of an unscarred uterus is associated with significant morbidity and mortality. Schrinsky and Benson [5], in their study, found a maternal and fetal mortality rate of 20.8% and 64.6%, respectively.

Because of inadequate obstetric services, our patient had a sonographic examination 18 hours after the first symptoms. Fortunately, the uterine pedicle was intact and there was no more bleeding.

## 4. Conclusion

The most common cause of uterine rupture is the presence of a uterine scar. Measures aimed at reducing the high maternal and perinatal mortality and morbidity associated with uterine rupture include health education of the masses, proper antenatal care, early referral of at-risk patients, and supervised hospital delivery. Importance should be given to the pain symptoms that can guide the diagnosis especially in a woman with no particular history.
